# X-ray free-electron laser wavefront sensing using the fractional Talbot effect

**DOI:** 10.1107/S1600577519017107

**Published:** 2020-02-12

**Authors:** Yanwei Liu, Matthew Seaberg, Yiping Feng, Kenan Li, Yuantao Ding, Gabriel Marcus, David Fritz, Xianbo Shi, Walan Grizolli, Lahsen Assoufid, Peter Walter, Anne Sakdinawat

**Affiliations:** a SLAC National Accelerator Laboratory, 2575 Sand Hill Road, Menlo Park, CA 94025, USA; b Argonne National Laboratory, 9700 South Cass Avenue, Lemont, IL 60439, USA

**Keywords:** wavefront sensing, free-electron lasers, FELs, interferometry, X-ray optics

## Abstract

Wavefront sensing at X-ray free-electron lasers is important for quantitatively understanding the fundamental properties of the laser, for aligning X-ray instruments and for conducting scientific experimental analysis. A fractional Talbot wavefront sensor has been developed. This wavefront sensor enables measurements over a wide range of energies, as is common on X-ray instruments, with simplified mechanical requirements and is compatible with the high average power pulses expected in upcoming X-ray free-electron laser upgrades. Single-shot measurements were performed at 500 eV, 1000 eV and 1500 eV at the Linac Coherent Light Source. These measurements were applied to study both mirror alignment and the effects of undulator tapering schemes on source properties. The beamline focal plane position was tracked to an uncertainty of 0.12 mm, and the source location for various undulator tapering schemes to an uncertainty of 1 m, demonstrating excellent sensitivity. These findings pave the way to use the fractional Talbot wavefront sensor as a routine, robust and sensitive tool at X-ray free-electron lasers as well as other high-brightness X-ray sources.

## Abstract 2

A wavefront sensor for an X-ray free-electron laser has been developed using the fractional Talbot effect. This wavefront sensor enables measurements over a wide range of energies, as is common on X-ray instruments, and is compatible with the high average power pulses expected in upcoming X-ray free-electron laser upgrades.

## 1. Introduction   

As X-ray free-electron lasers (FELs) (Kim *et al.*, 2017 ▸; Pellegrini *et al.*, 2016 ▸; Attwood & Sakdinawat, 2017 ▸) and synchrotron sources (Attwood & Sakdinawat, 2017 ▸; Eriksson *et al.*, 2014 ▸) continue to evolve with the ability to produce more distinctive X-ray beam properties, X-ray wavefront sensing (Chalupský *et al.*, 2010 ▸; David *et al.*, 2011 ▸; Idir *et al.*, 2014 ▸; Keitel *et al.*, 2016 ▸; Rutishauser *et al.*, 2012 ▸; Kayser *et al.*, 2016 ▸; Berujon *et al.*, 2015 ▸) becomes increasingly important for gaining a fundamental understanding of these beams and providing the required feedback for their manipulation. Two common wavefront sensors (WFS) currently being utilized at FELs are ones based on the Talbot grating interferometer (Pfeiffer *et al.*, 2005 ▸; Rutishauser *et al.*, 2012 ▸; Kayser *et al.*, 2016 ▸; Assoufid *et al.*, 2016 ▸; Matsuyama *et al.*, 2012 ▸; Liu *et al.*, 2018 ▸), which have primarily been used for hard X-rays, and ones based on the Hartmann wavefront sensor (Idir *et al.*, 2014 ▸; Keitel *et al.*, 2016 ▸), which have primarily been used in the extreme ultraviolet regime. Systematic wavefront measurements at different locations of the beam – for example, after the undulator, after transport mirrors and in the instruments – lead to a better understanding of beam imperfections and enable feedback for targeted corrections and improvements. Studies on changes in the FEL source wavefront under different accelerator configurations provide input into FEL tuning, including new methods in machine-learning (Edelen *et al.*, 2018 ▸), and enable pathways for more optimal operation. Similarly, accurate knowledge of the wavefront allows feedback to the optics for proper alignment, diffraction-limited focusing and sample-beam alignment.

At present, XFEL facilities are being upgraded to offer new capabilities to users. One major upgraded parameter is the repetition rate. For example, in the Linac Coherent Light Source (LCLS) upgrade to LCLS II, the planned repetition rate for soft X-ray beams will increase by almost 10 000 times, from 120 Hz to 1 MHz (LCLS, 2019 ▸). The resulting high average power leads to new challenges for survivability of beamline instrumentation, including the WFS. In addition, practical implementation of the WFS at either an XFEL or synchrotron facility requires coverage over a wide range of energies while minimizing mechanical motion and footprint. Again, using LCLS II as an example, photon energies in the range 250–1500 eV are available at its soft X-ray end-stations, and a WFS needs to cover this entire energy range while maintaining a limited footprint and minimal mechanical motion that are compatible with available instrument space. The fractional Talbot WFS addresses both requirements.

The following sections describe the design of the fractional Talbot WFS and report both simulation and experimental results on achieving full spectrum (250–1500 eV) coverage with minimal distance adjustments. Experiments were conducted at the Atmoic, Molecular and Optical science (AMO) Instrument at LCLS to verify the validity of the design. We demonstrate the capability and versatility of the fractional Talbot WFS through experiments to monitor beamline optics adjustments and to perform accelerator tapering studies.

## 2. Design and simulation of the fractional Talbot WFS   

**Figure 1:**
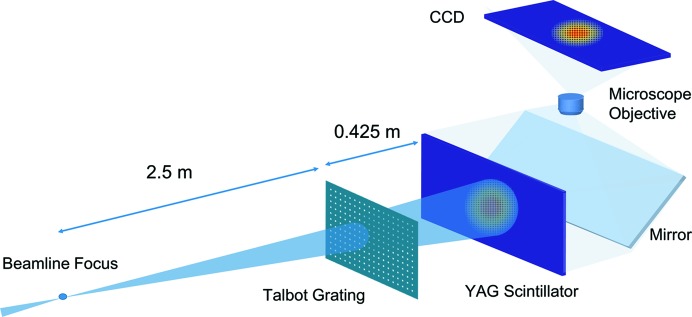
Schematic of the wavefront sensor in simulation, which consists of a 2D grating, followed by a YAG scintillator and a visible-light microscope. In simulation, the 2D grating has a period of 30 µm and a duty cycle of 1:10, with 3 µm-wide square openings. The grating is located 2.5 m from the beamline focus, and the YAG scintillator is 0.425 m from the grating.

The design of the fractional Talbot WFS is guided by the two requirements of survivability and mechanical constraints. The WFS consists of a grating mask and a scintillator-based detector. The X-ray beam is incident on the grating mask, which produces self-images in defined planes which are then imaged by the scintillator-based detector. A schematic of the fractional Talbot WFS setup is shown in Fig. 1 ▸.

A WFS based on the Talbot grating interferometer (Matsuyama *et al.*, 2012 ▸; Assoufid *et al.*, 2016 ▸; Liu *et al.*, 2018 ▸) utilizes the self-image of a periodic structure, typically a grating, to measure the amplitude and phase of the wavefront. This method has recently been demonstrated with FEL radiation (Liu *et al.*, 2018 ▸) on a single-shot basis with high sensitivity and accuracy in both the soft and the hard X-ray region. Additionally, it is compatible with coherent or partially coherent beams, focused or unfocused beams, and works on a wide spectrum of photon energies. However, the Talbot distance, *Z*_T_ = 2*p* ^2^/λ, where *p* is the pitch of the phase grating and λ is the wavelength of the light, is inversely proportional to the wavelength. This distance requires the Talbot WFS to be adjusted as a function of the wavelength. However, adjusting the Talbot distance to accommodate such a wide range of photon energies becomes impractical, due to both space limitations and operational complexity.

The fractional Talbot effect (Siegel *et al.*, 2001 ▸) refers to the self-imaging of periodic objects at distances that are *m*/*n* times the Talbot distance *Z*_T_, where *m* and *n* are coprime. An example of utilizing the fractional Talbot effect for wavefront sensing occurs in the hard X-rays regions, where a phase grating typically with a 1:2 (open hole size:pitch) duty cycle is used (Rutishauser *et al.*, 2012 ▸; Assoufid *et al.*, 2016 ▸; Liu *et al.*, 2018 ▸). Because of the phase grating, there are phase modulations but no intensity changes at integer Talbot planes, whereas Talbot images can be recorded at a series of special fractional Talbot planes, for example *Z*_T_ = *sp* ^2^/8λ when a π-phase-shift checkerboard grating is used (Rutishauser *et al.*, 2012 ▸; Liu *et al.*, 2018 ▸). In such a setup, the fractional Talbot images all have the same pitch of *p*/2 and a constant duty cycle of 0.5, regardless of the chosen order *s*.

In this design, we further utilize the potential of the fractional Talbot plane by choosing different combinations of *m* and *n*. With this approach, a fixed distance can correspond to different fractional Talbot planes for different wavelengths, allowing wavefront measurements at various wavelengths with the same setup. Depending on the values of *m* and *n*, the Talbot images for different wavelengths will have different pitches. Regardless of the images’ pitches, the Talbot images are all shearing interferograms, from which the phase of the wavefront can be retrieved by using the same Fourier-based procedure. In addition, the availability of fractional Talbot planes at distances much smaller than the integer Talbot planes also allows implementation of the Talbot WFS in a constrained space at FEL or synchrotron end-stations. When using Talbot WFS to monitor a focused beam, the wavefront curvature of the focused beam will push the Talbot plane to a further distance. The minimal distance from focus to the Talbot image detector is 4*Z*_T_, when the grating is located at 2*Z*_T_ from the focus (see Section 4).

To design the WFS for survivability in a high average power beam, we consider the most vulnerable part of the WFS to radiation damage, the scintillator crystal. Compared with a direct CCD detector, a scintillator followed by an optical microscope offers better spatial resolution and can accept more X-ray photons before reaching saturation. However, since all soft X-ray photons will be absorbed in the thin layer (<1 µm) of the scintillator, heat dissipation is limited, and the scintillator will not be able to survive the accumulated temperature increase under a beam with high repetition rate and full pulse energy. Calculations show that, at 1 MHz repetition rate with 1 mJ pulse energy, the commonly used yttrium aluminium garnet (YAG) based scintillator will be damaged (Burian *et al.*, 2015 ▸) by a little more than 1% of the full energy X-ray beam. To protect the scintillator, the grating mask needs to block 99% of the beam. A diamond substrate more than 100 µm-thick is expected to withstand the full beam (with reasonable beam size) due to its exceptional thermal properties. To transmit only 1% of the X-ray beam, the 2D grating mask should have a duty cycle of 1:10 (open-hole size:pitch) or smaller. Fabrication methods for this type of 2D grating mask on diamond substrates are under development.

For example, given a grating mask thickness of 100 µm or more, holes with a 3 µm diameter can be reliably fabricated by etching (see Section 4) and correspond to an aspect ratio of 30:1. The 1:10 duty cycle requirement would put the grating pitch at a minimum of 30 µm. At a photon energy of 1500 eV or a wavelength of 0.83 nm, the first Talbot distance would be 2.2 m, which requires a minimum 8.8 m distance from the WFS scintillator to the main experimental chamber. Such a geometry would be impractical for soft X-ray end-stations, where space is limited and vacuum chambers are required. Using fractional Talbot planes allows a much more compact and practical setup. Our design accommodates the full setup within 3 m and thereby offers flexibility in implementation and no interference to normal operation in the main chamber.

**Figure 2:**
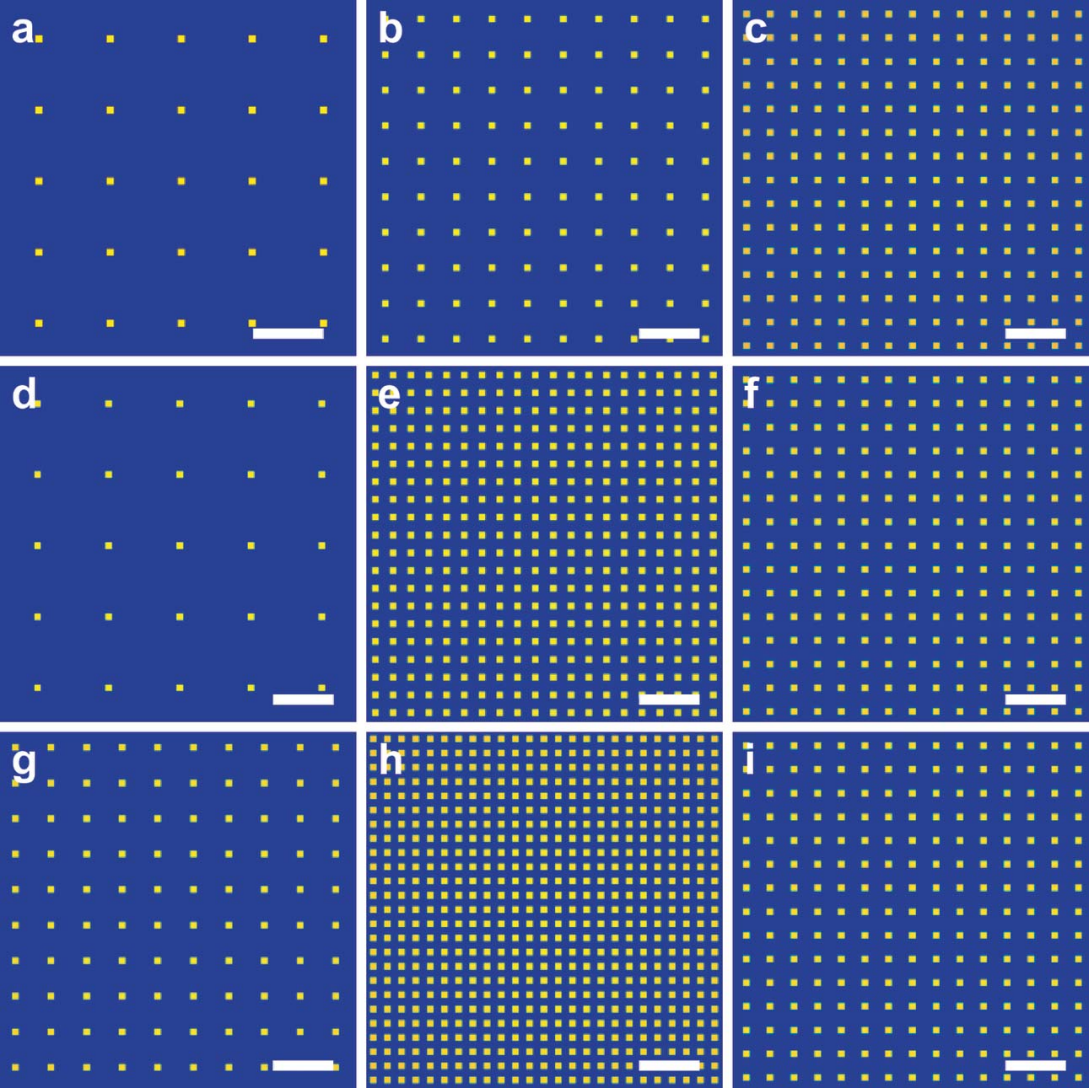
Simulated fractional Talbot images at different photon energies. The fractional Talbot planes offer a pathway to cover a wide spectrum in Talbot interferometry WFS. When using a 1:10 duty cycle, 30 µm pitch grating placed (*a*) 2.500 m from the focus, photon energies at 333.3 eV, 375 eV, 500 eV, 666.7 eV, 750 eV, 1000 eV, 1250 eV and 1500 eV will all form sharp Talbot images at a fixed location, 0.425 m from the grating, corresponding to 3/4, 2/3, 1/2, 3/8, 1/3, 1/4, 1/5 and 1/6 fractional Talbot planes, shown in (*b*)–(*i*), respectively.

In this simulation, we use a 2D binary amplitude grating with a 30 µm pitch and an open square hole size of 3 µm [Fig. 2 ▸(*a*)]. The grating is placed 2.5 m from a 1 µm (full width at half-maximum) focus, and the grating movement is restricted to a relatively short travel range of ±25 mm in the direction of the optical axis to allow for alignment and tuning to the operation wavelength. The YAG scintillator is placed at a fixed distance of 2.925 m from the focus. Figs. 2 ▸(*b*)–2(*i*) show the simulated fractional Talbot images obtained at photon energies of 333.3 eV, 375 eV, 500 eV, 666.7 eV, 750 eV, 1000 eV, 1250 eV and 1500 eV, corresponding to the fractional planes 3/4, 2/3, 1/2, 3/8, 1/3, 1/4, 1/5 and 1/6, respectively.

**Figure 3:**
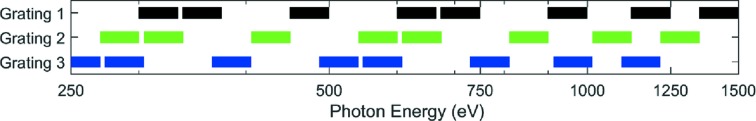
Photon energies for the three gratings at the LCLS II end-station. The whole spectrum from 250 eV to 1500 eV can be covered using multiple fractional planes; three gratings with pitches of 30 µm (grating 1), 31.6 µm (grating 2) and 33.3 µm (grating 3), and a short travel range (±25 mm) stage adjustment. Notice the very limited spectral coverage available from the stage adjustment alone, denoted by the width of each bar. The full spectral coverage is mainly enabled by the use of multiple fractional Talbot planes.

The whole spectrum from 250 eV to 1500 eV can be covered by the combination of these fractional planes using three interchangeable gratings with different pitches, namely 30 µm, 31.6 µm and 33.3 µm, and the tuning offered by the ±25 mm travel range of the grating. Fig. 3 ▸ shows how these factors work together to seamlessly cover the whole spectrum. The availability and utilization of the fractional planes are the most dominant factors to achieve this coverage. Because of the abundance of various fractional planes, coverage overlaps at some energies. In addition, we required perfectly ‘on-focus’ Talbot images in the simulations. In practice, Talbot images can tolerate a *z*-displacement or a slightly off-tuned wavelength. Although this would cause a blur in the images, the Fourier-based analysis is not generally affected by a small amount of blur. It has been shown that radiation with 10% relative bandwidth has little impact on the phase retrieval results in a grating interferometer (Momose *et al.*, 2017 ▸). Therefore, we expect more freedom on choosing the pitches of the gratings and/or appropriate fractional planes and more flexibility on the geometries in real operation.

## 3. Experimental results   

**Figure 4:**
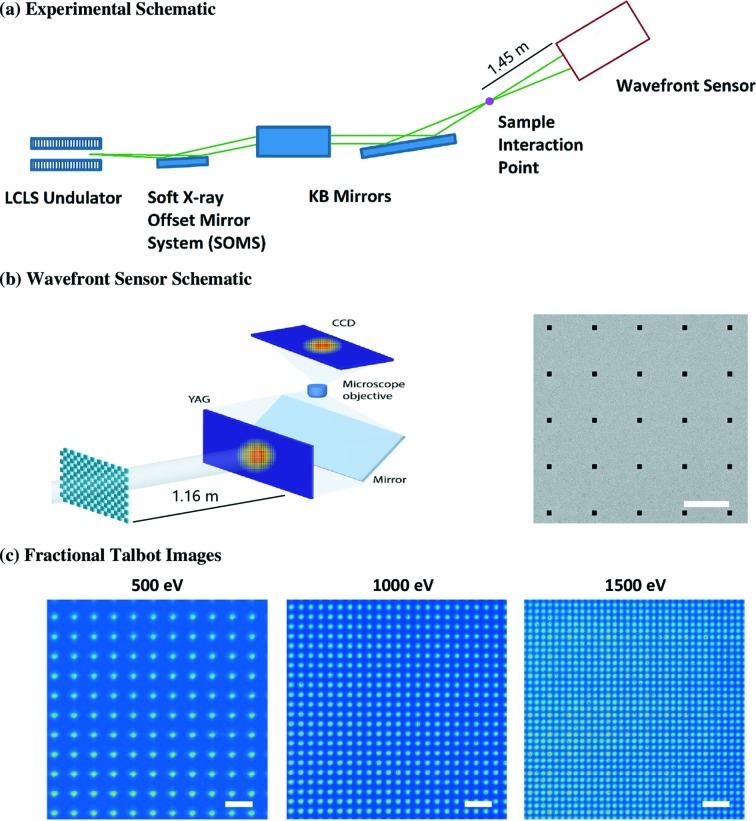
(*a*) Experimental layout at the AMO beamline of LCLS. The fractional Talbot wavefront sensor was placed after the sample interaction point. (*b*) Schematic of the wavefront sensor, which consists of a grating located 1.45 m from the interaction point, followed by a YAG scintillator located 1.16 m from the grating and a visible-light microscope. A scanning electron microscopy image of the silicon grating fabricated is shown to the right of the schematic. The 2D grating has a period of 40 µm and a duty cycle of 1:10, with 4 µm-wide square openings. Its 1% transmission limits heat load on the YAG scintillator to prevent damage (scale bar: 40 µm). (*c*) Recorded fractional Talbot images at 500 eV (left), 1000 eV (middle) and 1500 eV (right); scale bars are all 100 µm.

The fractional Talbot WFS was implemented at the AMO instrument at LCLS (Osipov *et al.*, 2018 ▸). To optimize the setup for this instrument, the fractional Talbot WFS was implemented using a 2 mm × 2 mm area silicon grating with 4 µm × 4 µm open squares at a 40 µm pitch (1:10 duty cycle) and a thickness of 100 µm (see Section 4). A schematic of the setup is shown in Figs. 4 ▸(*a*) and 4(**b**). We utilized an existing secondary chamber to host the grating, placing the grating 1.45 m from the interaction point. The YAG scintillator was then placed at an additional 1.16 m beyond the grating. With this geometry, the corresponding fractional Talbot planes for 500 eV, 1000 eV and 1500 eV were 1/2, 1/4 and 1/6, respectively, which correspond to the simulations. These three energies were chosen to verify the capability of the WFS for wide spectrum coverage.

### 3.1. Confirmation of multiple fractional Talbot images with a fixed setup   

In the first part of the experiment, we confirmed the existence of the fractional Talbot images using a fixed setup with the geometry mentioned earlier. As shown in Fig. 4 ▸(*c*) ▸, fractional Talbot images at 500 eV, 1000 eV and 1500 eV were successfully recorded on a CCD with 2048 × 2048 pixels in a visible-light microscope which magnified the images on the YAG onto the CCD. The microscope zoom lens was adjusted to cover a large enough field of view for all photon energies while at the same time resolving the densest period at 1500 eV sufficiently. Under such conditions, the effective pixel size (at the YAG) is 1.4 µm. The pitches of the Talbot images change from 72 µm for 500 eV, to 36 µm for 1000 eV, and to 24 µm for 1500 eV as expected.

In previous work, we showed that the 2D wavefront and the 3D reconstruction, through free-space propagation, of the FEL beam can be retrieved from such Talbot images (Liu *et al.*, 2018 ▸). Accurate determination of absolute wavefront aberrations, including astigmatism and higher-order distortions, requires calibration of the wavefront sensor to remove systematic errors, using, for example, a near-perfect reference wave generated from a spatial filter, as we demonstrated with hard X-rays at LCLS. Calibration of this WFS using a pinhole filter will be implemented at soft X-ray end-stations during the LCLS II upgrade. In this experiment, we performed relative measurements where systematic errors canceled. In the next two subsections, we report the results of such relative wavefront measurements on KB mirror adjustments and undulator source studies.

### 3.2. Sensitivity to Kirkpatrick–Baez mirror adjustment   

Besides full reconstruction of the light field, a more routine use of the WFS is to determine the exact location of the beam focus and use this as feedback for mirror adjustments. In particular, most beamlines employ a Kirkpatrick–Baez (KB) focusing system where focusing in the horizontal and vertical directions is provided by two independent mirrors. In a misaligned KB system, the aberration is dominated by astigmatism when the two mirrors do not focus to the same plane. A sensitive WFS monitoring small focus drifts in both directions is essential for beamline optic alignment.

**Figure 5:**
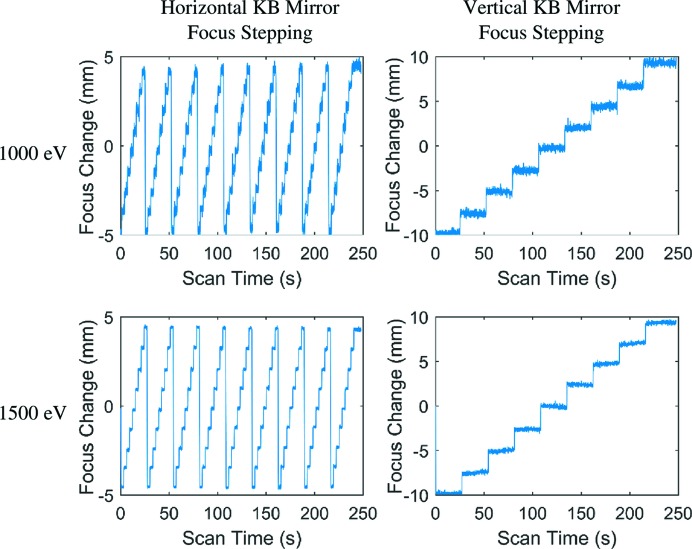
Sensitivity of fractional Talbot WFS to KB mirror adjustments. WFS retrieved effective focus location changes in the horizontal (left column) and vertical (right column) directions when the KB mirrors were step-scanned following commands in a 2D scanning script. In each set of scans, the vertical KB mirror was kept at the same position before stepping its focus by ∼2 mm, while the horizontal mirror was scanned in ∼1 mm steps for any given vertical mirror position. The same WFS was used for measurements at 1000 eV (top row) and 1500 eV (bottom row).

In the second part of this experiment, the sensitivity of the fractional Talbot WFS was tested by step-scanning the focal lengths of the KB mirror pairs. In each set of scans, the vertical KB mirror was kept at the same position before stepping its focus by ∼2 mm, while the horizontal mirror was scanned in ∼1 mm steps for any given vertical mirror position. The scans use a beamline script that was previously determined from the calculated incident angle and curvature changes caused by the controlled KB mirror adjustment motors. Images were continuously collected on a single-shot basis during the scan. The wavefront radius of curvatures was determined in both the horizontal and vertical directions by measuring the precise pitches of Talbot images in the corresponding directions. When a grating is placed at distance *D* from a point source, and the detector is placed at distance *Z* from the grating, the pitch of the Talbot image is magnified to
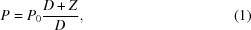
where 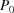 is the pitch of the Talbot image if the grating is illuminated by a plane wave (note that, for fractional Talbot images, 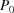 may not be the same as the original pitch of the grating). If the point source moves by *x* downstream (towards the WFS), the pitch of the Talbot image changes to 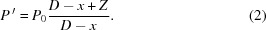
Therefore, by measuring *P* and *P*′, the change of source location can be determined as 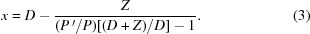
When *x* ≪ *D*, *Z*, equation (3) can be rewritten as 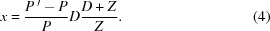
The sensitivity of the measurements of the focus change is then determined by the precision of the measurements of the relative pitch change. We selected a magnification of the microscope to ensure adequate sampling of the Talbot images, *e.g.* the smallest pitch of the Talbot image at 1500 eV still has more than 17 pixels per pitch. Sub-pixel resolution can be obtained by analysis in the Fourier domain, where averaging across many pitches further improves the precision. With this approach, we can capture a relative pitch change on the order of 10^−4^ (Liu *et al.*, 2018 ▸), primarily limited by photon shot noise, thus providing a very sensitive measurement on the focus position change. In this experiment, KB mirror scans at both 1000 eV and 1500 eV were performed (due to beam time limitation, such a scan was not performed at 500 eV) and the focus position changes, using the fractional Talbot images, were retrieved. For both vertical and horizontal directions, the corresponding one-dimensional lineouts were obtained by summing a box of 100 pixels near the center of the CCD to reduce the shot noise and calculate the pitches. As shown in Fig. 5 ▸, at both photon energies, the adjustments of the KB mirrors are clearly detected and the measured steps are in quantitative agreement with the set values by the beamline script. The noise level of detection, calculated from the standard deviation of the determined focus positions at 1500 eV when the KB mirrors are kept at the same position, is σ = 0.12 mm. This result confirmed the high sensitivity of this technique. At 1500 eV, a change of focus by 0.12 mm with a nominal focus at 1450 mm away corresponds to a root-mean-squared phase error of λ/150 in the 1 mm field of view of the Talbot image. For comparison, the Rayleigh length, 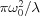, for the 1 µm nominal focus (ω_0_ = 0.4 µm) at 1500 eV is 0.6 mm.

Note that the noise levels at 1000 eV are higher than 1500 eV. Two factors can contribute to this. First, the photon beam at 1500 eV has a smaller divergence angle than the beam at 1000 eV, as both are fully coherent FEL beams with the same nominal beam size at focus. At the WFS location, the 1500 eV beam is more concentrated; therefore, it has higher intensity and more CCD counts, and thus lower shot noise. Second, the fractional Talbot image pitch at 1500 eV is 1.5× smaller. Therefore, there are more periods available within the same field of view. Because the pitch measurement is the average of all periods, this reduces the noise level further. Thus, by zooming out to a larger field of view we should be able to collect more signal from 1000 eV images, and bring the noise level lower, approximately to the same level as currently obtained for 1500 eV. Moreover, the biggest contributor to the shot noise in all cases is the grating with only 1% transmission. Note that this choice is to ensure that the scintillator will survive under the high average power of the planned LCLS II upgrade, which is ∼100× higher than the current LCLS level. Employing new scintillator materials, or operating under lower average power modes, could reduce this requirement and allow the use of higher transmission gratings, thus reducing the noise and improving sensitivity.

### 3.3. FEL source position effects due to undulator tapering   

**Figure 6:**
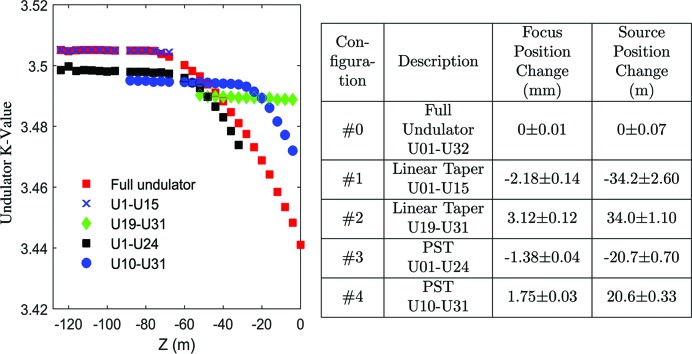
Measured relative focus and source position change under different undulator configurations. (Left) Different tapering of the undulators under each configuration. The normal operation mode, full undulator, is used as the reference point. Positive positions are further downstream along the beam path and negative positions are upstream. The results are statistics derived on running averages of the raw measurements from the experiments. Each raw measurement uses one single-shot image, and the running average uses 100 raw measurements. The uncertainties of the results are represented as ±1 standard deviation of all 10 000 measurements (after the running average) for each undulator configuration.

In the third part of the experiment, the WFS was used to study the fluctuation in the FEL source position and changes under different undulator configurations. In typical FEL operation (the baseline configuration), LCLS uses 30 undulators. For a 500 eV photon energy, the FEL saturation point is approximately 75 m upstream of the undulator exit, or 185 m from the AMO station. In this experiment, four additional configurations were studied (see Fig. 6 ▸ and its table). The baseline configuration (#0) employs all the sections with a post-saturation tapering. The two shortest configurations (#1 and #2) had 14 and 13 active sections, respectively; this distance is roughly the length for the FEL photon beam to reach saturation. The other two longer configurations (#3 and #4) added 8 more active sections with post-saturation tapering (PST) (see Fig. 6 ▸), in which the *K*-values of the individual undulator sections gradually drop in discrete steps after the nominal saturation point. The choice of these configurations and the measurements of the corresponding wavefronts are part of an effort to (1) benchmark FEL simulation tools using a new performance metric in the linear growth regime, near saturation, and far into the post-saturation and nonlinear regime; and (2) provide insight into particle trapping dynamics in the post-saturation regime. These factors are critically important for increasing the FEL efficiency and pushing the peak FEL powers into the TW regime. Wavefront measurements on the FEL beams under the chosen configurations can provide direct information on photon beam properties under different FEL gain regimes (near, post and deep saturation) and provide experimental feedback to benchmark the modeling, and potentially to improve the FEL operation with better taper performance.

In all cases, the FEL was tuned to 500 eV with a 30 Hz repetition rate. The images were continuously captured with a 30 ms interval to obtain single-shot images. In each configuration, 10 000 images were continuously collected for reliable statistics. In the comparisons, the Talbot image pitches were calculated (in the horizontal direction) and converted into relative focus position changes at the interaction point by using the method described earlier. We then used the thin lens equation to estimate the changes in relative source position at the undulator, assuming a source location at 185 m and a KB focal length of 1.60 m (in the horizontal direction; see Section 4). The results are summarized in the table of Fig. 6 ▸. Note that the measurement uncertainties are photon shot noise limited, which are worst for the two short configurations, #1 and #2, where the pulse energy is only 4% of the full undulator case (0.06 mJ versus 1.5 mJ). A running average of 100 raw measurements (each measurement uses one image) greatly reduced the shot noise and resulted in a much-improved determination of the source locations, with an uncertainty of ∼1 m in most cases. The change in the relative source position agrees reasonably well with the expected values using the undulator lengths (4 m per unit) and physical displacements, for example, the difference of ∼68 m between #1 and #2, and the difference of ∼41 m between #3 and #4. The continuous advances of the equivalent source location along the propagation direction after the beam reaches saturation (*e.g.* by ∼14 m from #1 to #3, and by ∼20 m from #3 to #0) provides insight into the post-saturation tapering dynamics.

## 4. Methods   

### 4.1. Distances in the Talbot interferometer with point source illumination   

When a grating is placed at distance *R* from a point source, the curvature of the illumination will push the perfect self-imaging Talbot plane to a location at 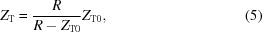
where *Z*_T0_ is the normal Talbot distance when using a plane-wave illumination. The total distance from the point source and the Talbot plane is thus 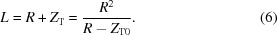
For a given *Z*_T0_, *L* will reach its minimal value of 4*Z*_T0_ when *R* = 2*Z*_T0_.

### 4.2. Grating fabrication   

The grating was fabricated by electron beam lithography. First, a 100 µm-thick silicon substrate was coated with a 200 nm Cr layer using electron beam evaporation. Then, a 1.3 µm-thick layer of polymethyl methacrylate (PMMA) electron beam resist was spun on the substrate and baked for 90 s at 180°C. Electron beam lithography (Jeol JBX 6300) was then used to pattern a 2 mm × 2 mm array of 4 µm × 4 µm open squares with a 40 µm pitch, giving a 1:10 duty cycle. The exposure was performed using an averaging scheme to minimize field stitching errors (Gleason *et al.*, 2012 ▸). After electron-beam exposure, the PMMA was developed at room temperature with 7:3 ratio of isopropyl alcohol:H_2_O for 55 s, rinsed in deionized water for 10 s and dried with nitrogen. The PMMA pattern was transferred to the Cr layer via Cl_2_O_2_ reactive ion etching (PlasmaTherm Versaline). The Bosch process was then used to etch holes through the 100 µm-thick Si substrate with the Cr pattern as the hardmask. An SEM image of the final grating mask is presented in the right-hand column of Fig. 4 ▸(*b*).

### 4.3. Converting measured wavefront curvature changes to undulator source location changes   

We use the thin lens equation in one dimension (the horizontal direction) to link the wavefront curvatures before and after the KB mirror as 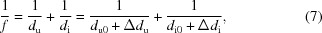
where *d*_u_ is the undulator source location, *d*_i_ is the focus location (both measured from the focusing mirror), and *f* is the effective focal length of the focusing mirror. We used nominal values: *f* = 1.60 m, *d*_u0_ = 185 m and *d*_i0_ = 1.61 m. For every determined focal position change (Δ*d*_i_) using the method described in Section 3.2, we used this equation to calculate the corresponding undulator source location change (Δ*d*_u_).

## 5. Conclusions   

A wavefront sensor using the fractional Talbot effect has been demonstrated with soft X-rays at the LCLS. With a fixed-distance setup, high sensitivity has been achieved at three photon energies: 500 eV, 1000 eV and 1500 eV. Small changes in the focal plane from KB mirror adjustments, on the order of 0.1 mm, and FEL source location changes as a function of various tapering schemes, on the order of 1 m, can both be detected by the sensor.

The utilization of the fractional Talbot effect will enable the practical implementation of routine operation of the Talbot wavefront sensor at FEL and synchrotron beamlines over a wide range of photon energies with minimal adjustments, both improving operation efficiency and reducing requirements on space and stages.
